# Use of standardized nasal and skin decolonization to reduce rates of bacteremia in patients undergoing extracorporeal membrane oxygenation

**DOI:** 10.1017/ash.2025.9

**Published:** 2025-02-12

**Authors:** Madyson Taylor, Russell L Griffin, Jeremey Walker, Catina James, Angela Akinsanya, Mary Duncan, Rachael A Lee

**Affiliations:** 1 Department of Medicine, University of Alabama at Birmingham, Birmingham, AL, USA; 2 Department of Epidemiology, University of Alabama at Birmingham, Birmingham, AL USA; 3 Department of Infection Prevention, University of Alabama at Birmingham, Birmingham, AL, USA

## Abstract

**Objective:**

We aimed to determine if implementation of universal nasal decolonization with daily chlorhexidine bathing will decrease blood stream infections (BSI) in patients undergoing extracorporeal membrane oxygenation (ECMO).

**Design:**

Retrospective cohort study.

**Setting:**

Tertiary care facility.

**Patients:**

Patients placed on ECMO from January 1, 2017 to December 31, 2023.

**Intervention:**

Daily bathing with 4% chlorhexidine soap and universal mupirocin nasal decolonization were initiated for all ECMO patients May 2021. The primary outcome was rate of ECMO-attributable positive blood cultures. Zero-inflated Poisson regression analysis was performed to estimate rate ratios (RRs) for the association between decolonization with BSI rates.

**Results:**

A total of 776 patients met inclusion criteria during the study period, 425 (55%) preimplementation and 351 (45%) post-implementation. Following implementation of decolonization, the overall incidence rate of BSI increased nonsignificantly from 10.7 to 14.0 infections per 1000 ECMO days (aRR 1.09, 95% CI 0.74–1.59). For gram-positive cocci (GPC) pathogens, a nonsignificant 40% increased rate was observed in the post-implementation period (RR 1.40, 95% CI 0.89–2.21), due mostly to a significant increase in the crude rate of *Enterococcus* BSI (RR 1.89, 95% CI 1.01–3.55). Excluding *Enterococcus* resulted in a nonsignificant 28% decreased rate (aRR 0.72, 95% CI 0.39-1.36) due to a nonsignificant 55% decreased rate of MRSA (aRR 0.45, 95% CI 0.18–3.58).

**Conclusions:**

Implementation of a universal decolonization protocol did not significantly reduce rates of certain BSIs, including MRSA and other gram-positive pathogens. Although nonsignificant, reduction in BSI rates in this patient population has important implications on surveillance metrics, such as MRSA, and in the future, hospital-onset bacteremia.

## Introduction

Within healthcare systems, patients can shed pathogens, and these pathogens can contaminate the hospital environment, allowing for opportunities to spread to other patients.^1^ Despite efforts to prevent transmission, hospitals across the United States are experiencing increasing rates of multidrug-resistant organisms (MDROs), including methicillin-resistant *Staphylococcus aureus* (MRSA). Decolonization with chlorhexidine (CHG) is superior to regular soap not only because of its antiseptic properties but also because it binds to skin proteins and continues to exert antiseptic activities for up to 24 hours.^2^ CHG-decolonization is also favored to reduce MDROs over the use of topical antibiotics alone or in combination with topical antibiotics.^3^

Hospitals have employed advanced modalities for caring for critically ill patients, including extracorporeal membrane oxygenation (ECMO), a machine similar to heart–lung bypass that utilizes cannula to provide oxygenation. Bloodstream infections are common in patients with ECMO, particularly patients colonized with MDROs such as MRSA, and preventing colonization and subsequent infection will reduce mortality.^4^ The duration of ECMO support affects risk of infection, particularly for patients with greater than 10 days on ECMO support.^5,6^

Studies have shown that universal decolonization with daily CHG bathing and intranasal mupirocin of patients in the intensive care unit (ICU) resulted in a significantly greater reduction in blood stream infections (BSI) than targeted decolonization or screening and isolation.^7^ In noncritical units, daily chlorhexidine bathing for all patients plus mupirocin for known MRSA carriers led to a 32% reduction in all-cause bacteremia and a 30% reduction in cultures of MRSA in patients with medical devices.^7^ Further, in a post-hoc analysis of a large scale cluster randomized trial, CHG bathing significantly reduced not only MRSA infections, but also all cause bloodstream infections.^2^ To our knowledge, only 2 studies have assessed the effect of decolonization on rates of bacteremia in ECMO, with no data on if these efforts improve rates of bacteremia due to specific MDROs.^7,8^ Given high rates of bacteremia, particularly MRSA, in ECMO patients, we implemented the use of daily chlorhexidine bathing and nasal MRSA decolonization in May of 2021. Prior to implementation, patients did not receive nasal decolonization and the amount of chlorhexidine was not standardized. We hypothesize that a CHG bath and intranasal mupirocin decolonization program for ECMO patients will significantly reduce both MRSA and other bloodstream infections.

## Methods

A quasi-experimental trial was conducted of all patients admitted to University of Alabama at Birmingham (UAB) Hospital and placed on ECMO from December 2017 to December 2023. Daily bathing with 4% chlorhexidine soap and universal nasal decolonization with mupirocin were initiated for all patients initiated on ECMO beginning May 2021, following the AHRQ guidelines for universal ICU decolonization.^9^ Preimplementation period was defined as any patient initiated on ECMO between December 2017 and April 2021. Prior to May 2021, no ICU utilized nasal decolonization and CHG bathing was not standardized. In May 2021, we implemented a standardized bathing and nasal decolonization protocol in our ECMO unit. Post-implementation period was defined as any patient initiated on ECMO June 2021-December 2023. Patients initiated on ECMO during the washout period or crossed over from preimplementation to post-implementation were excluded. Staff were educated according to standard protocols for CHG bathing and nasal mupirocin.^10^

The primary outcome was rate of ECMO-attributable positive blood cultures. Bloodstream infection (BSI) was defined as positive blood cultures with a clinically relevant pathogen identified in one or more cultures occurring 2 or more days after ECMO cannulation. For coagulase negative *Staphylococcus*, true bacteremia was defined as culture growth from two or more blood cultures obtained within 2 calendar days of one another. Pathogens were categorized as gram-positive cocci (GPCs), gram-negative rods (GNRs), and candidemia. For the GPC group, subcategories were created for MRSA, methicillin sensitive *Staphylococcus* aureus (MSSA), coagulase-negative staphylococci (CNS), and *Enterococcus* species pathogens. For each pathogen and category, only the first true bacteremia was included. Blood cultures were performed by the ECMO team only if there was a concern for infection. There were no surveillance blood cultures obtained and no antimicrobial prophylaxis in ECMO patients. Follow-up time ended at the time of first BSI for a given pathogen category or cessation of ECMO, whichever occurred first. Other data collected included baseline patient characteristics and clinical data, unit days and ECMO days, microbiology data, interventions for source control, dates of line placement, and clinical outcomes (ie, length of stay, mortality). ECMO cannulation type was defined by the original cannulation type; any change in type was not included.

Categorical variables were analyzed with either Fisher exact test or chi-square analysis when appropriate, and continuous variables analyzed with the t test or Wilcoxon rank-sum test when appropriate. Zero-inflated Poisson regression analysis with the natural log of ECMO days as the offset was used to estimate rate ratios (RRs) and associated 95% confidence intervals (CIs) for the association between daily bathing and nasal decolonization with BSI rates overall and by pathogen. Least absolute shrinkage and selection operator (LASSO) process was used to select appropriate parameters for the BSI models. Adjusted models included as covariates: COVID-19 infection, season of the year, prior history of myocardial infarction, cardiovascular disease, chronic obstructive pulmonary disorder, and renal disease; the logit process for the zero-inflated Poisson model included the count of blood cultures taken. In a secondary analysis, due to literature reporting a high risk of *Enterococcus* infection among COVID patients, the all-pathogen BSI and GPC rates were examined in the zero-inflated Poisson models excluding *Enterococcus*-positive cultures.^11,12^ A p-value <.05 was considered significant, and SAS v9.4 was used for all analyses.

## Results

A total of 776 patients met inclusion criteria during the study period. Of these, 425 (55%) were designated as preimplementation and 351 (45%) as Post-implementation (Table 1). Demographic data was overall similar between the two implementation groups, including sex, race, age, and Charlson comorbidity score. More patients with MI (35.1% vs 27.1%, *P* = 0.02) and moderate-severe liver disease (3.5% vs 1.1%, *P* = 0.04) were identified in the preimplementation group, whereas more mild liver disease was identified in the post-implementation group (9.4% vs 15.4%, *P* = 0.01).

**Table 1.** Demographics based on implementation group

	Total N = 776	Pre N = 425	Post N = 351	P-value
**Demographics**				
Sex, (%)				
Female	277 (35.7)	159 (37.4)	118 (33.6)	0.27
Male	499 (64.3)	266 (62.6)	233 (66.4)	
Race				
White	463 (60.0)	260 (61.2)	201 (58)	0.57
Black/African American	255 (32.9)	136 (32.0)	119 (33.9)	
Hispanic/Latino	13 (1.7)	8 (1.9)	5 (1.4)	
Other/refuse	45 (5.8)	21 (4.9)	24 (6.8)	
Age, mean (SD)	49.3 (16.4)	48.8 (17.0)	50.0 (15.8)	0.30
Age >65 years, (%)	148	81 (19.1)	67 (19.1)	0.99
History myocardial infarction, (%)	244 (31.4)	149 (35.1)	95 (27.1)	**0.02**
Congestive heart failure, (%)	519 (66.9)	282 (66.4)	237 (67.5)	0.73
Peripheral vascular disease, (%)	229 (29.5)	114 (26.8)	115 (32.8)	0.07
Dementia, (%)	4 (0.52)	4 (0.9)	0 (0.0)	0.13
Coronary artery disease, (%)	175 (22.6)	92 (21.6)	83 (23.6)	0.55
Chronic obstructive pulmonary disease, (%)	205 (26.4)	110 (25.9)	95 (27.1)	0.74
Rheumatic disease, (%)	32 (4.1)	19 (4.5)	13 (3.7)	0.72
Peptic ulcer disease, (%)	24 (3.1)	13 (3.1)	11 (3.1)	1.00
Mild liver disease, (%)	94 (12.1)	40 (9.4)	54 (15.4)	**0.01**
Moderate-severe liver disease, (%)	19 (2.5)	15 (3.5)	4 (1.1)	**0.04**
Diabetes without complication, (%)	246 (31.7)	142 (33.4)	104 (29.6)	0.28
Diabetes with complication, (%)	59 (7.6)	35 (8.2)	24 (6.8)	0.50
Hemi-paraplegia, (%)	45 (5.8)	26 (6.1)	19 (5.4)	0.76
Renal disease, (%)	251 (32.4)	141 (33.2)	110 (31.3)	0.59
Malignancy, (%)	24 (3.1)	12 (2.8)	12 (3.4)	0.68
Metastatic tumor, (%)	5 (0.6)	3 (0.7)	2 (0.6)	1.00
HIV, (%)	3 (0.4)	1 (0.2)	2 (0.6)	0.59
Charlson comorbidity score, Mean (SD)	3.6 (2.8)	3.6 (2.8)	3.5 (2.8)	0.76
**Outcomes**				
ECMO type, (%)				
Venovenous	339 (44.2)	183 (43.9)	156 (44.6)	0.88
Venoarterial	428 (55.8)	234 (56.2)	194 (55.4)	
ECMO days, mean (SD)	17.3 (26.9)	16.5 (23.8)	18.3 (30.3)	0.36
Total LOS, mean (SD)	40.6 (45.4)	39.7 (45.3)	41.7 (45.7)	0.53
LOS (death excluded), mean (SD)	N = 383 49.8 (47.4)	N = 204 46.9 (40.6)	N = 181 53.2 (53.9)	0.20
Positive BAL culture, (%)	103 (13.3)	52 (12.2)	41 (14.5)	0.40
BAL culture with MRSA, (%)	21 (2.7)	13 (3.1)	8 (2.3)	0.66
Tested COVID-19 positive, (%)	70 (9.0)	35 (8.2)	35 (10.0)	0.45
Death while inpatient, (%)	391 (50.4)	221 (52.0)	170 (48.4)	0.32

*Estimated from a Fisher’s exact test or a t-test for categorical and continuous variables, respectively.

**Definitions of all diseases in demographics follow the current definitions described in the Charlson Comorbidity Index.

Patients were placed on ECMO for similar durations (16.5 days vs 18.3 days, *P* = 0.36). A total of 103 (13%) patients developed a bloodstream infection while on ECMO; among those who developed a BSI, there was no difference in time to BSI between the pre (median 16, IQR 9-34 days) and post (median 13, IQR 9–26 days) periods (Wilcoxon p-value = 0.2834).

Gram-positive cocci were the most often cultured pathogens for both the pre- (8%) and post-implementation (11.4%) periods, followed by Gram-negative rods (5.4% and 6.3% for pre- and post-implementation, respectively) and candidemia (4.5 vs 5.4%) (Table 2). Within each organism category, the most often cultured pathogens were *Enterococcus faecalis* for Gram-positive cocci, *Pseudomonas aeruginosa* and *Klebsiella pneumoniae* for Gram-negative rods, and *Candida albicans* and *Candida parapsilosis* for candidemia. When assessing cases of bacteremia by pathogen, there were more patients in the post-implementation group to have *Enterococcus* BSI (4% vs 7%, *P* = 0.03). (Table 2).

**Table 2.** Cultured pathogens of first positive culture (by organism type) among patients placed on extra corporeal membrane oxygenation

	Total (n = 776)	Pre (n = 425)	Post (n = 351)
**Candida species**			
**N**	**38 (4.9)**	**19 (4.5)**	**19 (5.4)**
*Candida albicans*	18 (47.4)	9 (47.4)	9 (47.4)
*Candida dublinensis*	1 (2.6)	3 (15.8)	1 (5.3)
*Candida glabrata*	5 (13.2)	–	2 (10.5)
*Candida kefyr*	1 (2.6)	–	1 (5.3)
*Candida krusei*	2 (5.3)	–	2 (10.5)
*Candida lusitaniae*	2 (5.3)	1 (5.3)	1 (5.3)
*Candida parapsilosis*	6 (15.8)	4 (21.1)	2 (10.5)
*Candida tropicalis*	2 (5.3)	1 (5.3)	1 (5.3)
*Candida* species unspecified	1 (2.6)	1 (5.3)	–
**Gram-negative rods**			
**N**	**45 (5.8)**	**23 (5.4)**	**22 (6.3)**
*Achromobacter (Alcaligenes) xyloxid*	1 (2.2)	1 (4.4)	
*Citrobacter youngae*	1 (2.2)	–	1 (4.6)
*Enterobacter cloacae complex*	5 (11.1)	3 (13.0)	2 (9.1)
*Escherichia coli*	3 (6.7)	–	3 (13.6)
*Klebsiella (Enterobacter) aerogenes*	2 (4.4)	2 (8.7)	
*Klebsiella oxytoca*	5 (11.1)	4 (17.4)	1 (4.6)
*Klebsiella pneumoniae*	10 (22.2)	5 (21.7)	5 (22.7)
*Morganella morganii*	1 (2.2)	–	1 (4.6)
*Proteus mirabilis*	2 (4.4)	1 (4.4)	1 (4.6)
*Pseudomonas aeruginosa*	13 (28.9)	6 (26.1)	7 (31.8)
*Stenotrophomonas maltophilia*	2 (4.4)	1 (4.4)	1 (4.6)
**Gram-positive cocci**			
**N**	**74 (9.5)**	**34 (8.0)**	**40 (11.4)**
*Enterococcus faecalis*	23 (31.1)	7 (20.6)	16 (40.0)
*Enterococcus faecium*	8 (10.8)	3 (8.8)	5 (12.5)
*Enterococcus gallinarum*	1 (1.4)	–	1 (2.5)
Methicillin-resistant *Staphylococcus aureus*	20 (27)	13 (38.2)	7 (17.5)
*Staphylococcus aureus*	9 (12.2)	5 (14.7)	4 (10.0)
*Staphylococcus capitis*	2 (2.7)	1 (2.9)	1 (2.5)
*Staphylococcus epidermidis*	9 (12.2)	3 (8.8)	6 (15.0)
*Staphylococcus lugdunensis*	2 (2.7)	2 (5.9)	–

*Percentages expressed as percent of overall population (for N rows) and, for specific organisms, percent of positive cultures of the specified organism type

**Figure 1.**
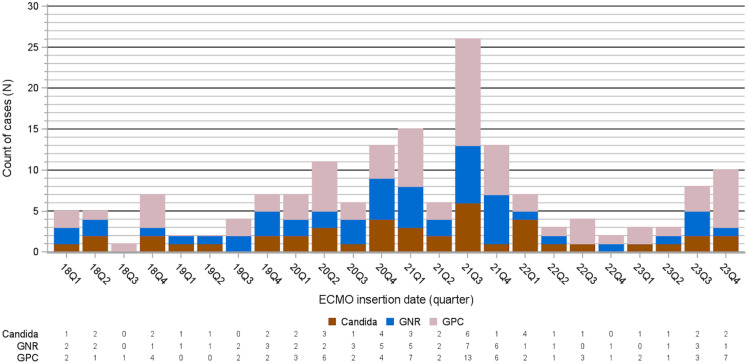
Count of blood stream infections by pathogen type (gram-positive cocci [GPC], gram-negative rod [GNR], and candidemia) and quarter. The first full quarter with daily bathing with 4% chlorhexidine soap and universal nasal decolonization with mupirocin was 21Q3.

Following implementation of daily CHG and nasal decolonization, the overall incidence rate of BSI increased from 10.7 to 14.0 infections for 1000 ECMO days; however, this increase was not significant either in crude analysis (RR 1.30, 95% CI 0.92-1.86) or adjusted models (RR 1.09, 95% CI 0.74–1.59) (Table 3). For GPC pathogens, a nonsignificant 40% increased rate was observed in the post-implementation period (RR 1.40, 95% CI 0.89–2.21), an association due mostly to a significant increase in the crude rate of *Enterococcus* BSI (RR 1.89, 95% CI 1.01–3.55), specifically an increase of *Enterococcus* that occurred during the third quarter of 2021 (Figure 1). An infection prevention investigation was performed but did not find a causative source of the cluster of cases of *Enterococcus faecalis.* Excluding *Enterococcus* from the GPC BSI rate resulted in a nonsignificant 28% decreased adjusted rate (RR 0.72, 95% CI 0.39–1.36) was observed; this association was driven by nonsignificant decreases rate of MRSA (adjusted RR 0.45, 95% CI 0.17–1.19) and MSSA (RR 0.81, 95% CI 0.18–3.58). Nonsignificant decreases were also observed for GNR rates (adjusted RR 0.84, 95% CI 0.48–2.18).

**Table 3.** Rate ratios (RRs) and associated 95% confidence intervals (CIs) for the comparison, overall and by pathogen, of risk and rate (per 1,000 ECMO days) of bloodstream infection between eras of daily bathing with 4% chlorhexidine soap and universal nasal decolonization with mupirocin

	Pre N = 425	Post N = 351	Crude RR (Post vs Pre) (95% CI)	Adjusted† RR (Post vs Pre) (95% CI)
**Total BSI**				
Rate	10.7 (8.3–13.9)	14.0 (11.0–17.9)	1.30 (0.92–1.86)	1.09 (0.74–1.59)
Excluding enterococcus	9.4 (7.2–12.3)	10.1 (7.7–13.3)	1.08 (0.73–1.59)	1.06 (0.72–1.57)
**Gram-positive Cocci**				
Overall Rate	5.6 (4.0–7.9)	7.9 (5.8–10.7)	1.40 (0.89–2.21)	1.17 (0.71–1.92)
Excluding *Enterococcus*	4.0 (2.7–5.9)	3.5 (2.3–5.4)	0.88 (0.49–1.59)	0.72 (0.39–1.36)
*MRSA*				
Rate	2.0 (1.2–3.4)	1.3 (0.6–2.6)	0.65 (0.27–1.56)	0.45 (0.17–1.19)
*MSSA*				
Rate	0.7 (0.3–1.7)	0.6 (0.2–1.7)	0.88 (0.24–3.26)	0.81 (0.18–3.58)
*CNS*				
Rate	1.0 (0.5–2.1)	1.3 (0.7–2.7)	1.31 (0.48–3.62)	1.15 (0.39–3.41)
*Enterococcus*				
Rate	2.4 (1.5–4.0)	4.6 (3.1–6.8)	1.89 (1.01–3.55)	1.72 (0.87–3.39)
**Gram-negative rods**				
Rate	3.6 (2.4–5.4)	3.8 (2.5–5.7)	1.06 (0.59–1.90)	0.84 (0.43–1.64)
**Candidemia**				
Rate	3.0 (1.9–4.7)	3.2 (2.0–5.0)	1.06 (0.56–2.01)	1.02 (0.48–2.18)

*Estimated from a zero-inflated Poisson.

†Adjusted for COVID infection, season of year, and prior history of myocardial infarction, cardiovascular disease, chronic obstructive pulmonary disorder, and renal disease; the logit process of the zero-inflated portion of Poisson model included count of blood cultures taken.

## Discussion

Implementation of daily CHG bathing and nasal decolonization with mupirocin protocol was not associated with changes in BSI rates overall or for any organism type (ie, candidemia, GNR, GPC). Of interest, though not significant, an increase in the *Enterococcus* BSI rate resulted in an overall increase in the GPC rate; however, GPC bacteremia rates were decreased once *Enterococcus spp.* were excluded.

Almost all studies investigating universal decolonization effect on MRSA and MDROs are in the ICU. One of the only studies, outside of this study, to investigate this question is a retrospective study of 3 French ECMO centers looking at a multiple-site decontamination regimen in VV-ECMO to see if this would decrease the rates of ECMO-acquired infections (defined as any infection acquired within 48 hours or more after admission) compared to patients that did not undergo this regimen.^8^ The regimen varied from the method seen in this study, including gut decontamination with antibiotics in addition to daily CHG bathing and nasal mupirocin.^8^ Patients receiving multiple-site decontamination had a lower incidence of ECMO-acquired infections and a statically significant decrease (*P* = 0.001) in MDRO infections.^8^ These results were mainly driven by reduction in ventilator associated pneumonia, with no difference in bacteremia rates, and highlight the need for similar multi-institution studies investigating decolonization efforts in ECMO units across the United States. Further support for the impact universal decolonization can have on MRSA acquisition is a study where universal decolonization was stopped in ICU patients.^13^ This hospital found a significant increase (*P* < .0001) in MRSA rates and bacteremia when there were no universal decolonization practices in the ICU compared with the prior use before discontinuation and the subsequent reintroduction of universal decolonization.^13^

One surprising point from our study was the increased incidence rates of certain pathogens causing BSI post-implementation. In our adjusted analysis, gram-positive pathogens such as coagulase negative *Staphylococcus* and *Enterococcus* were elevated in the post-intervention period. The identity of these other infections were limited in scope as many were co-infections with MRSA. The most common isolated infections included methicillin-sensitive *Staphylococcus aureus*, *Enterococcus faecalis*, *Klebsiella pneumoniae*, *Pseudomonas aeruginosa,* and *Candida albicans*, among a few other less common species. Almost all of these were not MDROs and could be treated with a variety of antibiotics. In addition, the increase in BSI was most prevalent in the third quarter of 2021. This included May 2021 when the decolonization protocol was initiated. Results during this month were considered washout infections and, therefore, not included in the data.

In patients with ECMO, increased rates of BSI have been described in the literature, especially with the bacteria implicated in this study. *Enterococcus spp*. have been associated with urinary tract infections, wound infections, and are commonly implicated in central line infections. Biofilm formation may also occur in ECMO lines, which are difficult to replace given patients are critically ill.^4^ Enterococcal BSIs have occurred at higher rates in other ICU centers during the COVID-19 pandemic and resulted in increased mortality.^11,12^ However, prior to COVID-19, early studies into CHG-cloth bathing in the ICU showed decreased the incidence of vancomycin-resistant Enterococcal (VRE) infections and less infections on patients’ and health care workers’ skin.^14^ The Vernon et all study identified VRE colonization and utilized contact isolation precautions in addition to CHG bathing. Our study’s Enterococci rate was measured only as BSI rate, which likely missed potential colonization cases and potentially did not measure the true effect of CHG bathing in this patient population. Given our research protocol, we did not assess intra-abdominal source for *Enterococcus spp.* BSI in this patient population. From an IP perspective, these patients were not located near each other in the unit or cared for by similar nursing staff. It is important to note that our study used 4% CHG liquid rather than the 2% CHG-impregnated cloth. CHG-impregnated clothes may provide lower bacterial densities with higher CHG concentrations.^15^ However, data is limited and the Rhee et al study is limited by the fact they only assessed healthy healthcare volunteers rather than assessing hospital patients while our study is investigating the effect of CHG bathing on a much sicker population. Finally, while chlorhexidine has been claimed to inhibit membrane-bound ATPase in *Enterococcus faecalis*, only high concentrations of chlorhexidine can collapse the membrane to cause its bactericidal effect.^16^ While we do not have data assessing bathing compliance in our ECMO population, it is important to note that re-education of staff to best practices for CHG bathing occurred in our institution in July 2022. This may have affected the improvement in *Enterococcus* rates given improvement in delivery of CHG on the skin.

An increase in BSIs and other hospital-acquired infections (HAI) during this time period in the COVID-19 pandemic was not limited to our institution. Many healthcare systems saw significant increases in HAIs during this time as well, with rates as high as a 60% increase in central line-associated BSI (CLABSI) and a 44% increase in MRSA bacteremia compared to pre COVID-19.^17,18^ Further, rates of MDRO and other BSI, including Gram-negative organisms, all increased with the surges of COVID-19.^17^ Similar increases in BSI, with a particular increase in MDROs, were seen in other single and multi-institutional studies across Europe during surges of the COVID-19 pandemic in both ICU and noncritical care settings.^19–21^ This trend is also reported with ECMO patients where 44% of 68 ECMO patients developed BSI, with Gram-positive organisms being the most common primary cause of a BSI and *Enterococcus faecalis* accounting for over half of those infections.^22^ These increases in BSI across levels of care and institutions provide support that the COVID-19 pandemic may have impacted the results of this study. This only increases the necessity for more studies around how decolonization strategies impact rates of BSI in ECMO patients. As the healthcare system is now in a post-COVID era of care, adherence to standard protocols can impact decolonization strategies and collaboration across institutions can evaluate the impact of decolonization on a larger patient population.

This study has some notable limitations. Given this is a retrospective study, confounding due to unmeasured data may occur, including identifying the true cause of bacteremia in this patient population. In addition, as a single institution study with a zero-inflated model, the study could potentially be underpowered. While the possibility that selection for resistant strains of organisms occurred therefore causing increased BSIs, the more likely culprit that impacted the results was the direct correlation with the COVID-19 pandemic. UAB hospital was heavily impacted by the COVID-19 pandemic and is the only hospital in the state with ECMO capability for adults. Similar to other hospitals, supply chain issues were present initially during the COVID-19, However, UAB hospital did not have supply chain issues with line care. The most likely impact of the pandemic in this study is that infection with COVID-19 led to longer hospitalization and ECMO days, both factors that increase patient risk for BSI. Individual bathing compliance may also confound our results. Bathing compliance has been measured since May 2022, with re-education in the unit in July 2022. These ECMO units have high overall measured CHG compliance at 94.7%, with the main unit having 11,806 patient days from May 2022- June 2024.

## Conclusion

Implementation of a universal decolonization protocol with daily CHG bathing and intranasal mupirocin reduced rates of MRSA bacteremia, although it was not significant. However, review of hospital onset bacteremia cases indicates improvement in MRSA bacteremia caused by ECMO. The lack of research in this field and the timing of this study almost exclusively assessing ECMO patients during the COVID-19 pandemic during the post-implementation period warrants further investigation on if decolonization strategies can reduce BSIs and MDRO bacteremia in ECMO patients. While there is extensive literature on epidemiology in ECMO and the risk factors for increased risk of infection, there is no standardization on what defines infection versus contamination, antimicrobial prophylaxis, and surveillance cultures, which is an issue due to the rapid expansion of ECMO care as a response to the COVID-19 pandemic.^4^ Despite these challenges, single center studies, such as this one, have shown promise in establishing more standards of care regarding prophylaxis, prevention, diagnosis, and treatment of ECMO-related infections. As ECMO care continues to become more prevalent around the world, the infectious disease community should continue investigation into effective prevention and treatment of ECMO associated BSI and establish standards for the care of these patients. Multi-institutional studies assessing the rates of BSI in ECMO patients with easily replicated compliance standards, consistent CHG-bathing techniques and longer follow-up times, including the post decannulation phase, should be conducted.
